# Interpreting fungal ecological contributions through taxonomic and functional profiling of metatranscriptomics

**DOI:** 10.3897/imafungus.17.185187

**Published:** 2026-04-29

**Authors:** Wenxing Hu, Jingfei Hu, Zhen Wang, Haihua Wang

**Affiliations:** 1 College of Plant Protection, Yangzhou University, Yangzhou 225009, Jiangsu Province, China College of Medicine, Linyi University Linyi China; 2 Dongshin University, Naju 58245, Jeollanam-do, Republic of Korea Dongshin University Naju Republic of Korea; 3 College of Medicine, Linyi University, Linyi 276000, China Department of Soil, Water, and Ecosystem Sciences, University of Florida Gainesville United States of America; 4 Department of Soil, Water, and Ecosystem Sciences, University of Florida, Gainesville, FL, 32611, USA College of Plant Protection, Yangzhou University Yangzhou China

**Keywords:** Bioinformatic pipelines, mycobiome, taxonomic classification

## Abstract

Fungi are ubiquitous across nearly all habitats and play pivotal roles in maintaining ecological sustainability. Understanding the composition and functional roles of active fungal communities is essential for elucidating fundamental ecosystem processes. Fungal metatranscriptomics has emerged as a powerful approach for simultaneously characterizing the composition and *in situ* functional activities of mycobiomes in diverse ecosystems. However, the bioinformatic techniques and pipelines for metatranscriptomic analysis are still less developed to interpret fungal ecological functions in ecosystems. In this review, we critically synthesize recent technological advances and persistent challenges in the analysis of metatranscriptomic data. Focusing specifically on taxonomic and functional classification, we systematically categorize current workflows into two main computational strategies; assembly-based and alignment-based methods, according to their analytical pipelines and tool implementations. Standardized comparisons show assembly-based pipelines deliver higher taxonomic and functional resolution for fungal metatranscriptomics but require heavy computational resources, while alignment-based pipelines are fast and scalable with fungal-specific marker-based tools performing better, though the performance largely relies on the database comprehensiveness. Notably, major hurdles include fungal transcriptome complexity, database biases, and a lack of standardized analytical workflows, thereby limiting broader application and reproducibility. Looking forward, future progress hinges on integrating long-read sequencing, expanding fungal-specific genomic and functional databases, and leveraging artificial intelligence to overcome current limitations in annotation and classification. Collectively, these developments are crucial for transitioning fungal metatranscriptomics from a descriptive ecology to a predictive science.

## Introduction

**Figure 1.**
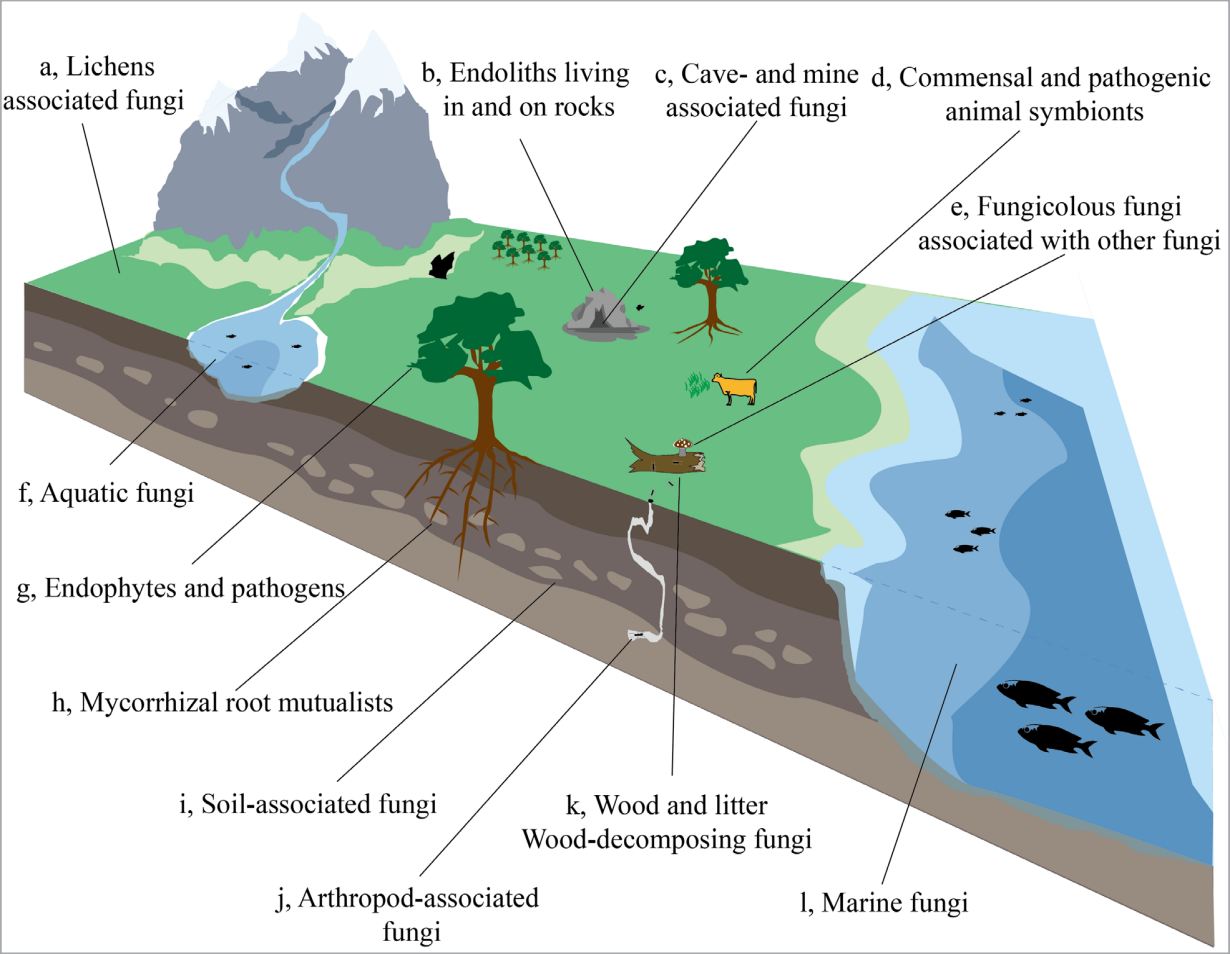
Diversity of the mycobiome habitats. Fungi are most commonly associated with terrestrial ecosystems, but can also be found growing on nearly any substrate on Earth, from deep ocean sediments to the animal gut.

Fungi represent one of the most diverse and widely distributed groups of organisms on Earth (Gonçalves et al. 2022). According to global assessment of fungal diversity, approximately 200,000 species have been formally described (http://www.speciesfungorum.org/; accessed 8 October 2025), while the estimated total number is thought to range from 2.2 to 3.8 million (Singh and Thakur 2023). They are distributed in nearly all habitats and maintain crucial roles in ecological functions (Fig. 1). In terrestrial and freshwater ecosystems, fungi play a central role in decomposing organic matter, driving biogeochemical cycles of key elements such as carbon, nitrogen, and phosphorus, and facilitating the mineralization of plant essential nutrients (Kijpornyongpan et al. 2025). For example, phosphate-solubilizing fungi can convert insoluble phosphorus into available forms for plant uptake (Duan et al. 2023). Moreover, through diverse plant-fungal interactions (e.g mycorrhizal symbioses, endophytic colonization, and pathogenic relationships), they significantly influence nutrient fluxes, carbon sequestration, and host plant performance across forest, grassland, and aquatic environments (Leake et al. 2004; Watkinson 2016). Consequently, precisely deciphering the composition and functional roles of active fungal communities in the environment is crucial for understanding fundamental ecosystem processes.

Although internal transcribed spacer (ITS) or rRNA amplicon sequencing has traditionally advanced fungal community studies, its effectiveness remains constrained by primer mismatches and variable PCR amplification efficiency (Li et al. 2020). For instance, universal primers such as ITS1F and ITS2 exhibit varying amplification efficiencies across fungal groups (e.g., *Ascomycota* versus *Basidiomycota*), potentially leading to biased estimates of community composition (Manter and Vivanco 2007). The varying rRNA gene copy numbers among fungal species, ranging from approximately 100–200 copies in *Saccharomyces cerevisiae* to over 500 copies in some *Basidiomycetes*, complicate fungal abundance estimation (Nilsson et al. 2019). Furthermore, amplicon sequencing fails to provide functional insights, thereby limiting our understanding of the *in situ* roles of fungi within ecosystems.

Metatranscriptomics offers a powerful alternative by directly capturing actively transcribed genes *in situ*, effectively addressing the above constraints (Cottier et al. 2018). Unlike amplicon sequencing, metatranscriptomics circumvents primer bias and, via RNA sequencing, directly reveals the RNA transcripts present under natural conditions (Shakya et al. 2019). For example, Schneider et al. (2021) studied the Norway Spruce rhizosphere ecosystem via metatranscriptomics and elucidated that *Cortinarius* fungi actively expressed transcripts related to the degradation of aromatic and heterocyclic organic macromolecules in natural conditions, a core metabolic function that could not be accurately identified by amplicon sequencing. Wang et al. (2023) integrated ITS amplicon sequencing with metatranscriptomics to explore ecological functions of *Dikarya* fungi in the Chinese coastal water ecosystem and revealed that planktonic fungi contributed more than 3% of eukaryotic carbohydrate-active enzyme (CAZyme) transcripts under natural water temperature gradients, disclosing their *in situ* function in mediating marine organic carbon degradation. Thus, metatranscriptomics allows for the simultaneous acquisition of taxonomic and functional information, enabling integrated analysis of “who is actively doing what” in a specific scenario. The capacity to directly link taxonomic identity with functional activity makes metatranscriptomics an exceptionally powerful tool for elucidating fungal ecological functions.

The rapid evolution of sequencing technologies provides crucial support for advancing metatranscriptomic studies. However, several challenges persist, including complex fungal transcriptomes, insufficient tools or reference databases, and a lack of standardized workflows (Shakya et al. 2019). This review systematically consolidates recent methodological advances and persistent limitations in mycological metatranscriptomics, with a dedicated focus on strategies for fungal taxonomic classification and functional annotation.

## Principles and pipelines for fungal metatranscriptomics

**Figure 2.**
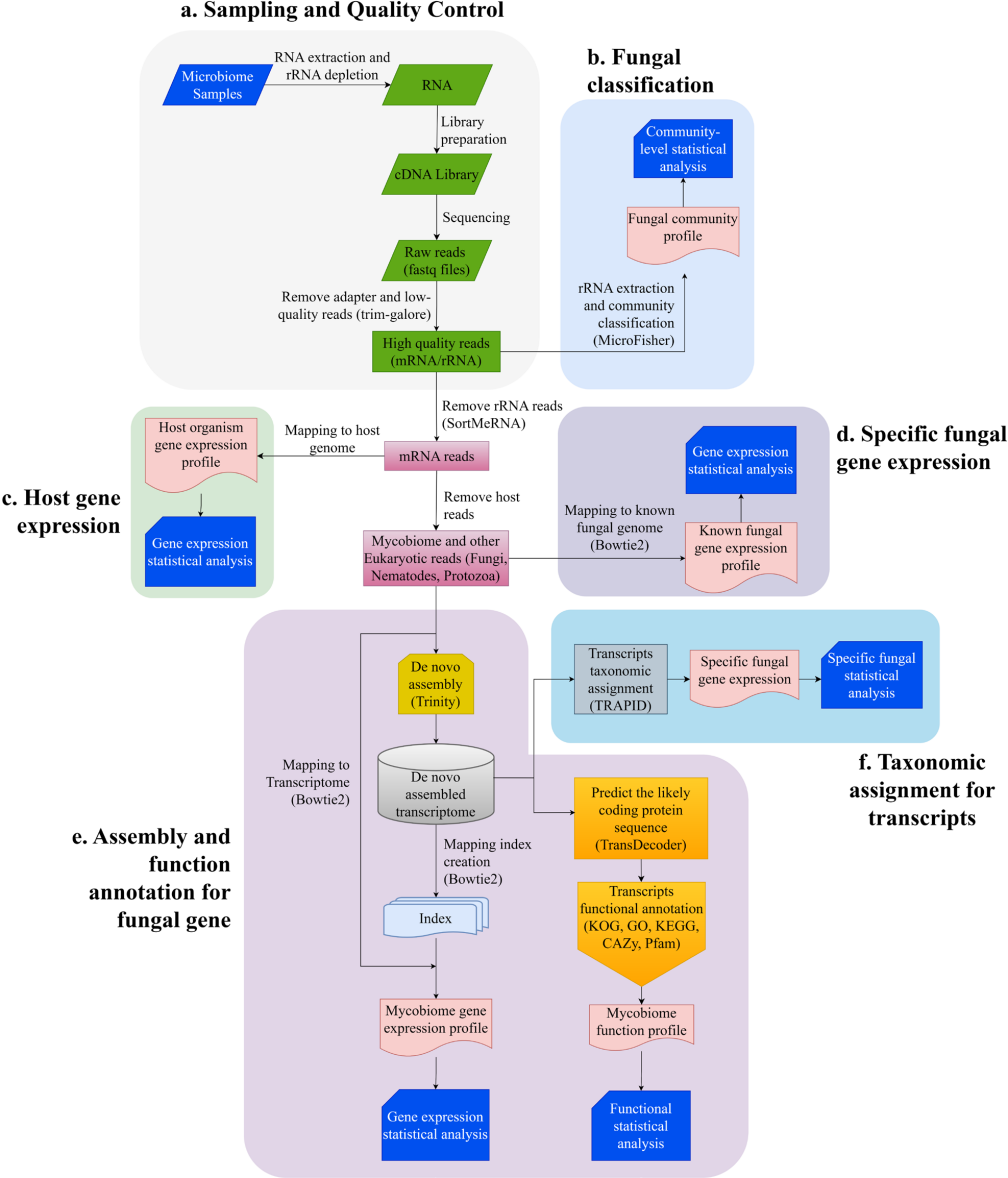
Pipeline for fungal metatranscriptomic data processing and interpretation. The workflow diagram comprises five modular components representing a comprehensive analytical pathway from raw sequencing reads to taxon-resolved functional inference.

Metatranscriptomics has emerged as a methodology with strong potential to simultaneously unravel taxonomic composition and molecular functions of fungal communities across diverse ecosystems (Auer et al. 2024). This integrated approach provides a comprehensive framework for characterizing the taxonomic and functional dynamics of mycobiomes, as illustrated in Fig. 2. A standard eukaryotic metatranscriptomic workflow starts with sample collection and total RNA extraction from environmental sources such as plant tissues, soil, litter, water, etc. (Mukherjee and Reddy 2020). Following extraction, ribosomal RNA (rRNA) depletion and poly-A enrichment are performed to enrich the eukaryotic messenger RNA (mRNA) fraction, which represents the pool of actively expressed genes (Zhao et al. 2014). The enriched mRNA is subsequently reverse-transcribed into complementary DNA (cDNA) for sequence library construction, with metatranscriptomic sequencing typically conducted on the Illumina platform. Raw sequencing data then undergo rigorous preprocessing steps, including quality control, adapter trimming, and removal of low-quality reads as well as potential host RNA sequences, to ensure the reliability of downstream analyses (Kumar and Yadav 2024). Using the resulting processed data (consisting primarily of residual rRNA and mRNA reads), custom bioinformatic pipelines are applied to achieve host-fungus sequence separation, taxonomic classification of fungal communities, and functional characterization of uncharacterized mycobiome members (Anwar et al. 2019).

Fungal taxonomic classification using metatranscriptomic data relies on two principal strategies, assembly- and alignment-based approaches. *De novo* assembly-based approaches primarily adopt the reads from either rRNA or other genes that are consistently transcribed without being susceptible to environmental stimulation. In practice, the target reads are first aligned and extracted through mapping the qualified dataset onto a comprehensive database containing reference sequences from all the accessible fungal species, such as the RDP and SILVA databases, to extract rRNA reads (Cole et al. 2014; Tedersoo et al. 2024). With extracted reads, a *de novo* assembly approach is applied to reconstruct full-length contigs. These sequences are subsequently classified through aligning against their corresponding reference databases followed by taxonomic identification and abundance quantification. Up to this point, the potential mycobiome community composition has been classified, while if precise phylogenetic placement is required, maximum likelihood or Bayesian algorithms can be employed to construct phylogenetic trees for determining taxonomic affiliations (Layola MR et al. 2025). Alternatively, aligning metatranscriptomic reads directly to well-constructed reference databases can allow for rapid taxonomic classification. This strategy enables efficient fungal species identification while bypassing computationally intensive assembly steps, and is utilized by most existing taxonomic classification tools for either metatranscriptomic or metagenomic analyses (Donovan et al. 2018). For instance, Wang et al. 2026 established a standardized approach for profiling fungal communities from metatranscriptomic data by directly aligning reads to hypervariable marker databases. Therefore, selecting an appropriate strategy is critical for achieving reliable and biologically meaningful characterization of fungal communities in metatranscriptomic studies.

To functionally annotate the fungal metabolic potential, non-host mRNA reads typically undergo *de novo* assembly to construct a comprehensive transcriptome. The sequences are then mapped to assembled transcriptome or known fungal genomes to reconstruct mycobiome expression profiles or known fungal gene expression profiles (Kuske et al. 2015). Simultaneously, the protein-coding potential of the assembled transcripts is predicted using tools such as TransDecoder, and the predicted proteins are functionally annotated against specialized databases (e.g., KOG, GO, KEGG, CAZy, Pfam) to reveal active metabolic pathways and functional traits (Blin et al. 2021). In addition, those assembled transcripts can be taxonomically assigned using platforms like TRAPID, which can directly link the functional activities to particular fungal species (Bucchini et al. 2021). This integrated approach addresses the critical question of “what are these active fungi doing?” by identifying species-specific expressed functional genes (carbohydrate-active enzymes, transporters, secondary metabolite synthetases, etc.) (Zeng et al. 2023). This provides powerful insights into the ecological roles and functional dynamics of the active fungal community (Bucchini et al. 2021). Notably, alignment-based functional characterization has been developed through directly aligning reads against the database compiled with function gene markers (Jung et al. 2013; Martinez et al. 2016). Although limitations exist, this approach has been widely employed in investigations on complex environmental samples, where sequencing depth is often limited in high-diversity communities, to explore the overall ecological functions of microbial communities.

Collectively, metatranscriptomic pipelines offer a powerful framework for understanding the composition and function of mycobiomes in diverse ecosystems (Tyagi and Katara 2024). Two principal bioinformatics strategies (assembly- and alignment-based) are typically employed. The assembly-based strategy involves *de novo* assembly of short sequencing reads into longer transcript sequences which provide more complete genetic information, thereby enhancing resolution and accuracy in subsequent taxonomic identification and functional annotation. An alignment-based strategy bypasses computationally intensive assembly steps by directly aligning preprocessed reads to existing reference databases, showing high computational efficiency and rapid processing. The selection between assembly-based and alignment-based strategies should be guided by specific research objectives, sample characteristics, and available genomic resources.

## Current bioinformatic strategies for taxonomic classification

**Figure 3.**
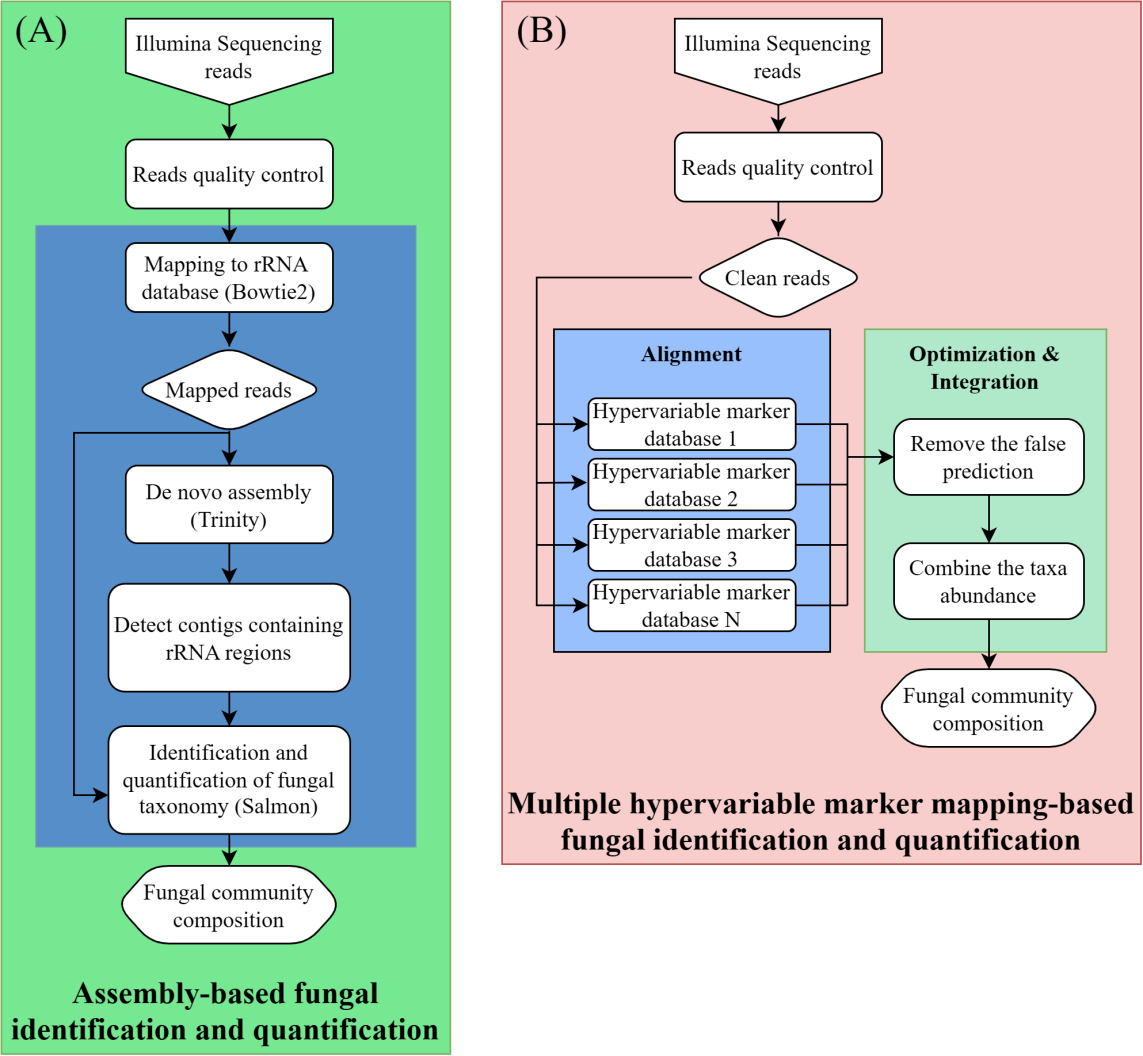
Comparative workflow of two metatranscriptomic pipelines for fungal community analysis. Schematic representation of two bioinformatic approaches for taxonomic classification of fungal communities from Illumina metatranscriptomic data: (**A**) Assembly-based pipeline involving de novo transcriptome reconstruction followed by taxonomic assignment of contigs; (**B**) Multi-marker mapping-based pipeline utilizing direct alignment of sequencing reads against fungal-specific hypervariable marker databases (Wang et al. 2026).

Taxonomic classification of metatranscriptomic data is primarily oriented toward fungal ecological function interpretation, which is essential for delineating the composition of active fungal communities and serves as the core link connecting fungal community composition to ecological processes. In practice, it clarifies the differentiation of fungal functional guilds, reveals the responses of fungal communities to environmental disturbances, and establishes robust links between fungal taxonomic composition and ecological processes. To achieve these goals, taxonomic analysis of metatranscriptomic data primarily relies on two bioinformatic strategies: assembly-based and alignment-based approaches. Each strategy has distinct advantages and limitations regarding underlying principles, tool performance, and applicable scenarios (Fig. 3).

### The assembly-based taxonomic classification

In assembly-based taxonomic classification, high-quality RNA-Seq reads are first extracted through mapping onto reference databases (e.g. RDP and SILVA databases for rRNA reads and specific databases for consistently transcribed mRNA reads) using alignment-based bioinformatic tools (e.g. bowtie2, STAR). With the extracted reads, *de novo* assembly methods are typically used to construct longer contigs for precise classification, using full length sequences of marker genes. Assembled sequences provide reference contigs of longer marker gene fragments, thereby facilitating homology-based searches against their corresponding reference, improving classification reliability, and enabling the discovery of novel species (Shakya et al. 2019). The selection of assemblers critically determines the completeness and accuracy of subsequent taxonomic assignments (Hölzer and Marz 2019). Commonly employed tools include those specifically designed for RNA sequencing, such as Trinity (Haas et al. 2013), Oases (Schulz et al. 2012), and rnaSPAdes (Bushmanova et al. 2019), while other tools including Trans-ABySS (Robertson et al. 2010), SOAPdenovo-Trans (Xie et al. 2014), and BinPacker (Liu et al. 2016) are also widely applied for *de novo* assembly of metatranscriptomic data (Table 1). The performance of these tools varies when processing fungal species with genomes that contain features such as alternative splicing, highly repetitive sequences, and complex gene families. Among them, Trinity is the most widely used transcriptome assembler. It employs a unique three-module architecture (Inchworm, Chrysalis, and Butterfly) and achieves notably high completeness in fungal gene recovery (Grabherr et al. 2011). Studies demonstrate that assembly with Trinity or Oases yields a greater number of annotatable transcripts compared to general assemblers, with Trinity exhibiting particularly high performance (Wang and Gribskov 2017). Furthermore, in a mouse gut metatranscriptomic dataset comprising 516,881 single-end reads, Trinity generated 21,454 annotatable contigs (BLASTX bit score > 50) and recruited 50.3% of raw reads to these contigs. In contrast, the generic metagenomic assembler MetaVelvet (k = 27) yielded only 13,706 annotatable contigs and mapped merely 32.8% of reads (Celaj et al. 2014). These results demonstrate that assemblers optimized for transcriptomic data consistently outperform generic metagenomic assemblers in fungal gene reconstruction.

**Table 1.** Comparison of bioinformatic tools for assembly, taxonomic classification, and abundance estimation of fungal mRNA transcripts.

Tool	Function	Features	Reference
Trinity	*De novo* transcriptome assembler	Reconstructs full-length transcripts without a reference genome. Recovers more complete transcripts across expression levels than many other assemblers.	(Grabherr et al. 2011)
Oases	*De novo* transcriptome assembler	Heuristically assembles RNA-seq reads which can handle alternative splicing. Improves on Trinity and Trans-ABySS in benchmarks.	(Schulz et al. 2012)
rnaSPAdes	*De novo* transcriptome assembler	Outperforms other tools by yielding more complete assemblies (in terms of gene and isoform counts) with concurrently higher accuracy. Built on the SPAdes genome assembler, optimized for RNA-Seq.	(Bushmanova et al. 2019)
Trans-ABySS	*De novo* transcriptome assembler	Extends ABySS assembler for RNA-Seq which assembles reads at multiple k-mer sizes and merges contigs. Identifies known, novel, and alternative structures within expressed transcripts during testing, demonstrating high sensitivity and specificity comparable to reference-based assembly methods.	(Robertson et al. 2010)
SOAPdenovo-Trans	*De novo* transcriptome assembler	Adapts SOAPdenovo for RNA data; designed for short reads. Provides higher contiguity, lower redundancy, and faster execution, as evidenced by its effective handling of practical challenges such as alternative splicing and variable expression levels.	(Xie et al. 2014)
BinPacker	*De novo* transcriptome assembler	Novel “bin-packing” approach to assemble isoforms. Incorporates coverage information to resolve isoforms; outperforms most other assemblers in tests, with low memory use.	(Liu et al. 2016)
MEGAHIT	Metagenome assembler	Ultra-fast *de novo* assembler for large (meta)genomic datasets. Not specific to transcripts (no splice handling), but sometimes used for mixed microbial assemblies.	(Buchfink et al. 2015; Li et al. 2015)
DIAMOND	Sequence aligner	Ultra-fast protein aligner. Supports high-throughput protein structure alignment with ultra-fast efficiency and high-sensitivity	(Buchfink et al. 2015; Pearson 2013)
BLASTX	Sequence aligner	Standard NCBI tool for translating nucleotide queries and aligning to protein databases. Widely used for transcript annotation via similarity searches.	(Pearson 2013)
BASTA	Taxonomy classifier	Command‐line lowest common ancestor (LCA) classifier. Takes BLAST/DIAMOND outputs and assigns sequences to taxa via customizable LCA rules regardless of length or sequencing platform.	(Kahlke and Ralph 2019)
TRAPID	Transcript Taxonomic assignment tool	Online system for *de novo* transcriptomes, ORF prediction, frameshift correction, gene family assignment, and functional and phylogenetic analysis.	(Bucchini et al. 2021)
Kallisto	Abundance estimation	For transcript or target sequence quantification, it employs pseudoalignment to directly determine read origin from RNA-Seq data without full-base alignment, streamlining the process for remarkable speed.	(Raghavan et al. 2022)
RSEM	Abundance estimation	An accurate and user-friendly software tool for quantifying transcript abundances from RNA-Seq data. Enables accurate transcript quantification for non-model organisms without a reference genome.	(Li and Dewey 2011)
Salmon	Abundance estimation	A dual-purpose tool that integrates alignment and quantification. it directly estimates transcript abundances from raw sequencing reads (FASTA/Q) using a transcriptome index in its quasi-mapping mode.	(Patro et al. 2017)

Following assembly, the reconstructed contigs can be taxonomically annotated through direct comparison against the corresponding target gene database. These include performing BLAST searches against comprehensive databases like NCBI followed by taxonomic assignment, or aligning sequences directly to curated fungal rRNA-specific databases such as SILVA and RDP for improved classification accuracy (Gweon et al. 2015). The classifiers, such as the tools BLASTn and RDP, integrated with specific set criteria determine the accuracy and precision of the taxonomic classification of assembled contigs. Multiple selection criteria, including e-value (expect value), length (sequence overlap), gapopen (number of gap openings), and mismatch (number of mismatches) for BLASTn, are recommended to accurately identify fungal taxonomy of contigs. Alternatively, for mRNA assemblies, functional annotation of mRNA transcripts via eggNOG-mapper can be integrated with platforms like TRAPID to infer taxonomic assignments (Bucchini et al. 2021). Abundance estimation of the classified fungal taxa can be performed through RSEM, kallisto, and salmon transcript quantification methods (Raghavan et al. 2022), then extracted reads can be directly mapped onto the constructed contigs reference.

The assembly-based taxonomic strategy substantially enhances taxonomic resolution and sensitivity (Tran and Phan 2020). Longer assembled sequences encompass a greater number of phylogenetically informative sites, enabling the differentiation of closely related species (e.g., different species within the same genus). For instance, Schneider et al. 2021 successfully identified over 95.5% of fungal transcripts at the phylum level and 34% at the species level in spruce rhizosphere samples by employing an assembly-based pipeline. Furthermore, the method holds unique value for discovering and characterizing novel or rare species absent from existing databases, as a full-length transcript without high-similarity matches in databases inherently suggests the presence of a new taxonomic unit (Workman et al. 2018).

However, several challenges remain. The substantial computational demands restrict its application in large-scale projects or resource-constrained environments (Warburton and Sebra 2023). Furthermore, transcripts from low-abundance species may fail to assemble, leading to a systematic bias toward dominant taxa (Freedman et al. 2021). Additionally, the prevalence of intron splicing and extensive alternative splicing in fungal genes poses significant challenges to assembly algorithms (such as MEGAHIT), frequently generating erroneous chimeric or fragmented transcripts that directly compromise the accuracy of subsequent taxonomic identification (Lindahl et al. 2013). These limitations collectively restrict the applicability of assembly-based classification. Thus, this strategy is most suitable for scenarios when deep sequencing is available and one seeks comprehensive, reference-independent profiling of the fungal transcriptome.

### The alignment-based taxonomic classification

The alignment-based approach directly matches sequencing reads to existing reference databases to achieve fast taxonomic classification, thus bypassing the computationally intensive assembly step. This approach relies on the completeness of the reference, which can be categorized as follows: 1) fungal genome based references (e.g., Kraken2, Centrifuge), which enable ultra-fast classification through optimized genome indexing for rapid sequence assignment (Kim et al. 2016a; Wood et al. 2019); 2) functional mRNA and rRNA marker gene-based methods (e.g., MicroFisher, MetaPhlAn4, EukDetect), which utilize species-specific marker genes for identification (Blanco-Míguez et al. 2023); and 3) protein family-based methods (e.g., FunOMIC-P), which achieve enhanced eukaryotic detection sensitivity through optimized protein sequence alignment and database construction (Table 2) (Xie and Manichanh 2022).

**Table 2.** Comparison of alignment-based bioinformatic tools for fungal taxonomic classification.

Tool	Category	Algorithmic Approach	Strength	Limitation	References
Kraken2	Genome-based (Genome database)	Exact *k*-mer matching with compact index	Extremely fast; high accuracy and scalable	Sensitive to database completeness and limited fungal coverage	(Wood et al. 2019)
Centrifuge	Genome-based (Genome database)	FM-index on concatenated sequences	Low memory use and fast	Limited fungal resolution and false positives	(Kim et al. 2016a)
MEGAN	Genome-based (NCBI or custom database)	BLAST/DIAMOND plus LCA assignment	Broad taxonomic profiling and visualization support	Requires pre-alignment; slower on big datasets	(Huson et al. 2007)
FunOMIC-P	Protein-based (Protein database)	Protein alignment for taxonomy and function	Functional and taxonomic profiling	Mainly human fungi; limited environmental coverage	(Xie and Manichanh 2022)
MetaPhlAn4	Marker-based (Prokaryotic DB)	Bowtie2 alignment to clade markers	Species-level abundances	Limited to taxa with defined markers	(Blanco-Míguez et al. 2023)
EukDetect	Marker-based (Curated fungal marker database)	Conserved eukaryotic protein markers	Sensitive to microbial eukaryotes	Misses novel fungi	(Lind and Pollard 2021)
MicroFisher	Marker-based (Fungal genomes)	Hypervariable ribosomal markers (ITS1, ITS2, LSU D1/D2)	High sensitivity and accurate	Very new; database expanding	(Wang et al. 2026)

These approaches exhibit variable performance in fungal classification from metatranscriptomics. Tools based on genomic databases, such as Kraken2 and Centrifuge, offer rapid processing speeds in taxonomic assignments; however, their sensitivity in detecting uncharacterized fungal species remains limited because fungal reference genomes are incomplete (Wood et al. 2019). Additionally, the use of short reads often results in insufficient taxonomic resolution at the species level (Portik et al. 2022). In practice, MetaPhlAn4 offers high specificity with minimal false positives by leveraging unique marker genes, albeit its detection range, and consequently its coverage of fungal diversity, is inherently limited by the composition of its predefined marker database (Blanco-Míguez et al. 2023). FunOMIC outperformed other tools in terms of recall and database comprehensiveness; however, its practical detection accuracy was limited by comparatively low precision (Avershina et al. 2025). EukDetect is specifically designed with a refined eukaryotic marker gene database, enabling superior detection of low-abundance eukaryotes in complex samples like gut and soil biotas and outperform generic tools in both sensitivity and specificity (Lind and Pollard 2021).

Comprehensive benchmarks for these tools on fungal metatranscriptomes are scarce, but evidence from similar work is informative. In an evaluation using synthetic mycobiomes, Avershina et al. (2025) found that performance in accurate classification of fungal communities from metatranscriptomics varied significantly across tools. General-purpose classifiers, such as Kraken2 and MetaPhlAn4, often produced false positives or failed to detect certain taxa. In contrast, specialized tools demonstrated superior performance; FunOMIC identified the majority of fungal species, while EukDetect produced community profiles that most closely resembled the true composition. Notably, the accuracy of marker-based tools like FunOMIC and EukDetect remained largely unaffected even when bacterial reads constituted 90–99% of the sample (Avershina et al. 2025). This suggests that marker-based methods can be robust to bacterial background noise, whereas k-mer classifiers may struggle when eukaryotic reads are scarce. In addition, due to the distinct target marker genes used by existing taxonomic classification tools, the fungal classification results may yield a significant difference. For example, Avershina et al. (2025) found only one fungus (*Candida orthopsilosis* Tavanti) was consistently detected by all tools across all simulated communities, emphasizing that no single method is universally reliable. Wang et al. (2026) compared the accuracy and precision of MicroFisher and other tools under a benchmarking condition, with results suggesting that MicroFisher and EukDetect outperform in fungal profiling from simulated metagenome data.

In metatranscriptome studies, alignment-based tools have been successfully applied under specific conditions. For instance, EukDetect enabled the discovery of eukaryotes overlooked by standard pipelines across various environments, including from the human gut, plant surfaces, and seawater (Lind and Pollard 2021). The newly developed tool, MicroFisher, employing the hypervariable marker regions of rRNA genes, demonstrated higher accuracy than Kraken2 or Centrifuge when tested on forest soil metatranscriptomic data (Wang et al. 2026). Kraken2 and Centrifuge have been utilized for rapid screening of potential fungal pathogens in clinical or food samples, though their results often require subsequent confirmation due to low specificity (Diao et al. 2023). In fungal microbiome investigations, tools originally designed for bacteria (e.g., Kraken2, MetaPhlAn4) are sometimes employed, but their results should be interpreted cautiously. Collectively, these benchmarking studies offer evidence-based recommendations for selecting appropriate analytical tools in practical research applications.

The primary advantages of alignment-based taxonomic assignment lie mainly in its superior computational speed with lower memory requirements (Liu et al. 2024). By circumventing the assembly process, it fundamentally eliminates biases introduced by assembly errors, thereby ensuring the accuracy and reliability of the results. However, this alignment-based strategy is highly dependent on the completeness of the reference database. For the majority of environmental fungi (e.g. soil saprotrophs, plant endophytes) that lack sequenced genomes, genome-based alignment methods either fail to identify them or can only assign them to related genera or families (Avershina et al. 2025; Wang et al. 2026). As complementary methods, the rRNA marker databases provide comprehensive and high species-specific resolution in fungal identification, relying on the worldwide contribution of rRNA genes over the last few decades (Cole et al. 2014). However, the limited genetic information carried by short reads often precludes precise species-level classification, particularly when targeting highly conserved genomic regions, typically resulting in taxonomic assignments only at the genus or family level (Schneider et al. 2021). Consequently, while this approach is unparalleled for rapid biodiversity surveys, its resolution and comprehensiveness are intrinsically constrained by both database limitations and the inherent properties of short-read sequences. Collectively, current fungal identification tools in taxonomic classification exhibit a trade-off between computational efficiency, taxonomic sensitivity, and precision, with performance being constrained by reference database completeness, marker gene selection, and read length.

### Strategy evaluation and selection guidelines

In conclusion, methodological selection should be guided by the specific goals of the research, the computational resources available, and the characteristics of the sample (Kuske et al. 2015). A clear understanding of these factors is crucial for choosing an appropriate research design that ensures the credibility and validity of the research findings (Li et al. 2018). The assembly-based strategy proves particularly advantageous for studies utilizing deep sequencing data and seeking to minimize reliance on existing reference databases, especially those aimed at complex community diversity assessment and potential discovery of novel taxonomic lineages. Conversely, the alignment-based strategy is suited for the rapid screening of large-scale cohorts, pathogen detection, or hypothesis-driven ecological studies. Its superior throughput and efficiency thereby make it particularly advantageous in epidemiological investigations and biodiversity monitoring programs.

Looking ahead, the development of fungal taxonomic tools is expected to mitigate the current limitations of fungal metatranscriptomics. First, the continued expansion and refinement of reference databases, driven by initiatives like the JGI 1000 Fungal Genomes Project, will incorporate genomic data from a wider array of environmental fungi. This integration will fundamentally enhance the potential of alignment-based strategies (Grigoriev et al. 2014). Second, applying a database that contains fungal-only or bacterial contamination-filtered sequences enables high-resolution and accurate taxonomy classification. Some pipelines use a hybrid strategy, for example, one might assemble transcripts and then run a fast classifier on contigs, or run BLASTn on all reads to triage abundant taxa and classification tools (MicroFisher, Eukdetect) on unclassified reads for sensitive detection (Lind and Pollard 2021). Finally, the application of machine learning holds unprecedented potential. Models capable of directly learning from sequence features to predict taxonomic placement will reduce reliance on traditional homology-based searches, thereby pioneering novel pathways for identifying unknown fungal lineages (Yan et al. 2025).

## Bioinformatic strategies for fungal functional characterization

**Figure 4.**
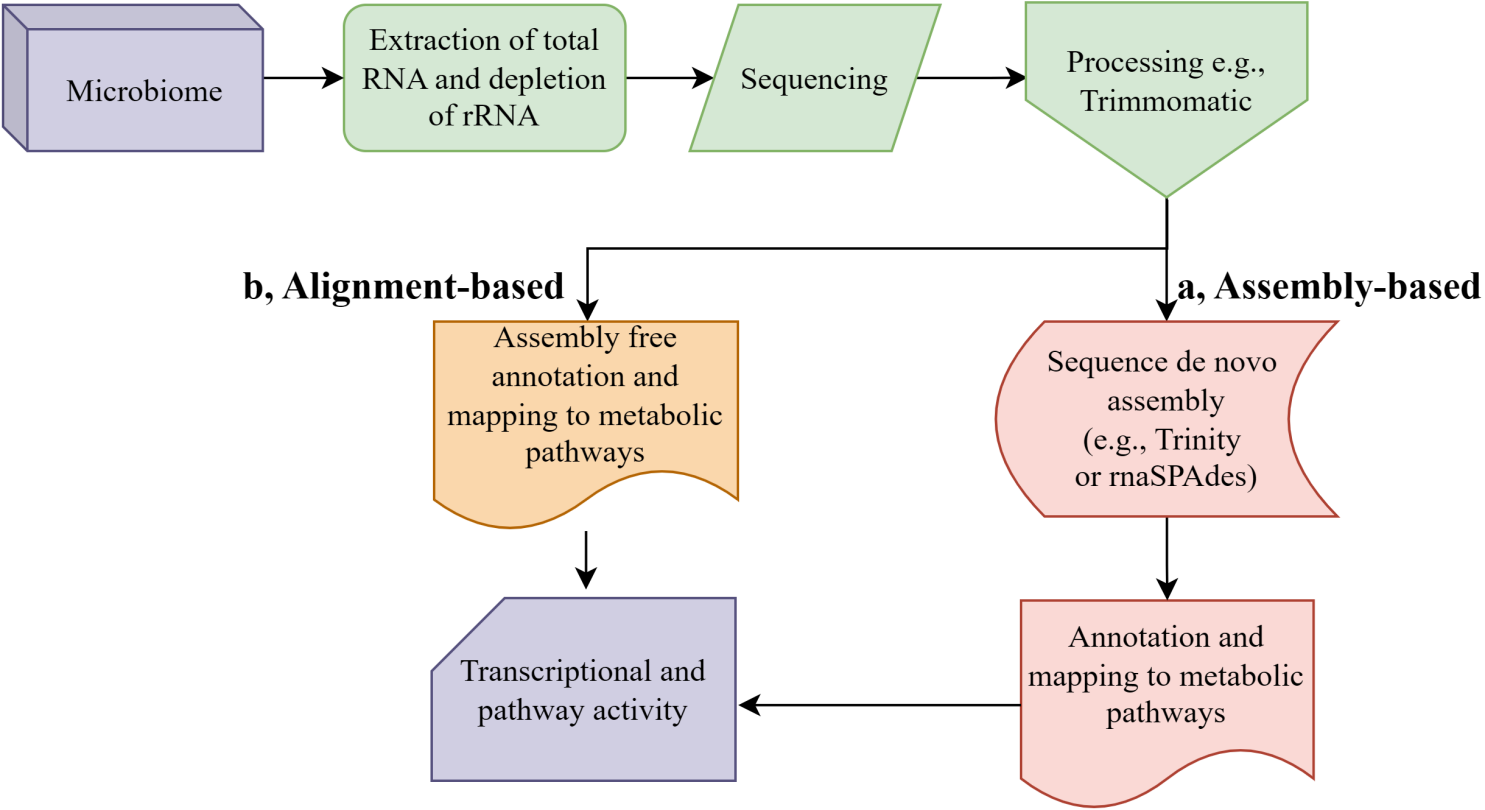
Workflow of metatranscriptomic analysis for fungal functional classification. Schematic representation of bioinformatic approaches for functional annotation, comparing two main strategies: (**A**) sequence reconstruction and functional classification based on de novo assembly, and (**B**) assembly-free functional profiling through direct read alignment to reference databases.

Functional classification elucidates the ecological functions of active fungal communities through directly addressing their transcriptional responses *in situ*. It involves annotating mRNA reads to reveal active pathways in metabolism, stress response, and nutrient cycling, thereby connecting taxonomic data directly to ecological processes (Law et al. 2022). Two methodological approaches are generally used, *de novo* assembly-based and read mapping-based functional classification strategies. Each possess distinct advantages and limitations in fungal functional assignment (Fig. 4).

### Assembly-based functional classification

The assembly-based strategy begins by reconstructing longer or complete transcript sequences using *de novo* assemblers such as Trinity or rnaSPAdes (Prjibelski et al. 2020), as described in section above. Subsequently, assembled transcripts are then processed through annotation pipelines for functional characterization. The transcript annotation compiles the mRNA gene and protein searches onto the reference databases, such as NCBI nt/nr, Pfam, InterproScan, and EggNOG (Table 3). Tools like TransDecoder are employed to predict open reading frames (ORFs) from the assembled transcripts (Brasseur et al. 2025; Mao et al. 2025). The predicted protein sequences and transcripts are then subjected to homology searches and domain analysis (via Pfam/InterProScan), followed by the assignment of Gene Ontology (GO) and KEGG terms to the respective transcripts (Ugarte et al. 2018). Integrated pipelines such as Trinotate combine multiple analytical approaches (BLAST, HMMER, and InterProScan) to simultaneously achieve protein domain identification, Gene Ontology (GO) term assignment, and KEGG pathway mapping (Hart et al. 2020). Similarly, eggNOG-mapper (Hernández-Plaza et al. 2023) can be applied to the assembled protein sequences to infer orthology-based functional categories, while InterProScan (Jones et al. 2014) effectively identifies conserved protein families. Reconstructed transcripts significantly enhance functional annotation by preserving full-length genes or large domains, thereby yielding more comprehensive functional insights compared to map raw reads (Ramberg et al. 2021). Afterward, the gene expression of assembled transcripts can be estimated using tools including RSEM, kallisto, salmon, and bowtie2.

**Table 3.** Bioinformatic tools for assembly-based functional annotation of fungal metatranscriptomic analysis.

Tool	Primary Role	Function	References
TransDecoder	Identification of protein-ORFs	Detects likely coding sequences in assembled transcripts using length, coding score, and domain support. Superior sensitivity and precision compared to current state-of-the-art methods in both reference transcripts and *de novo* transcriptome assemblies.	(Mao et al. 2025)
Trinotate	Transcript functional annotation	Integrates BLAST/DIAMOND, Pfam/InterProScan, GO, and KEGG assignments	(Hart et al. 2020)
eggNOG-mapper	Orthology-based annotation	Provides rapid and comprehensive functional annotation by mapping sequences to precomputed orthologous groups. scalable to large datasets	(Cantalapiedra et al. 2021)
InterProScan	Protein family and domain detection	Provides comprehensive functional characterization by simultaneously scanning protein sequences against multiple integrated databases (KEGG, MetaCyc, UniPathway, etc.).	(Jones et al. 2014)
antiSMASH plus SMURF	Secondary metabolite cluster annotation	New BGC comparison algorithm, allows for the integration of results from other prediction tools, and more effectively detects fungal biosynthetic gene clusters	(Blin et al. 2021; Khaldi et al. 2010; Musacchia et al. 2015)

Assembly-based functional profiling promotes effective correlation between gene expression and pathway activity, which has been widely used for molecular function classification. This strategy enables quantitative analysis of assembled transcripts across different experimental conditions, thereby inferring active metabolic pathways in specific environments. In practice, Hayden et al. (2018) identified numerous differentially expressed metabolic genes through assembly that were undetectable via raw-read mapping, including polyketide cyclases and terpenoid backbone enzymes in suppressive soils versus antibiotic biosynthesis genes in non-suppressive soils. Similarly, plant-fungal symbiosis research has successfully uncovered key fungal activities through assembly-based reconstruction. These applications collectively demonstrate how assembly-based annotation links fungal gene expression to ecological functions by providing complete gene sequences and enabling pathway activity quantification.

By directly linking functional activities to particular fungal species, the assembly strategy facilitates precise “who is doing what” functional associations by enabling the tracing of assembled transcripts back to specific fungal taxonomic units. For instance, Auer et al. (2024), using an assembly-based approach, demonstrated that saprotrophic fungi in soil significantly upregulate plant cell wall-degrading enzyme genes, while ectomycorrhizal fungi highly express nitrogen transporter genes, clearly elucidating the ecological function of the main fungal guilds in forest soils at refined taxonomic levels. To assign the taxonomic classification to the assembled transcripts, the most direct strategy is homology-based, which involves aligning transcripts to reference datasets (e.g., NCBI nt/nr, EggNOG databases) using tools like DIAMOND or BLASTX, with taxonomic assignments subsequently made by lowest common ancestor algorithms. For instance, Peng and Valentine (2025) employed a DIAMOND-BASTA-LCA pipeline, aligning metatranscriptomic sequences to the nr database to achieve classification at the fungal phylum level. Alternatively, marker-gene approaches scan for conserved single-copy genes within transcripts and match those to fungal clades. Integrating rRNA marker assignment with assembled mRNA transcript annotation significantly enhances overall classification confidence. For instance, Schneider et al. (2021) demonstrated that even with sparse reference genomes, combining ITS amplicon data with transcriptome assembly substantially improves the reliability of fungal classification.

Current assembly-based functional annotation tools remain constrained by database limitations. Although general-purpose resources (KEGG, UniProt, eggNOG, and Pfam) cover most core eukaryotic and prokaryotic genes, they exhibit significant underrepresentation of unique fungal genomic features such as secondary metabolite gene clusters (e.g., nonribosomal peptide synthetases and polyketide synthases) and carbohydrate-active enzymes involved in lignocellulose degradation. Similarly, fungal-specific functional modules including mycorrhizal symbiosis pathways and fungal hormone signaling pathways are not well annotated in those databases. While specialized tools like antiSMASH (Blin et al. 2021) and SMURF (Khaldi et al. 2010) enable targeted annotation of fungal secondary metabolite gene clusters, their dependence on genomic context limit their applicability in transcriptome-based annotation workflows. This database gap leads to erroneous annotations in homology-based searches, where non-fungal functions, such as calcium-dependent kinases or WRKY transcription factors, are incorrectly assigned to fungal proteins. To minimize misannotations arising from these limitations, it is recommended to integrate multiple databases, particularly specialized functional databases.

### Alignment based functional classification

The alignment-based strategy achieves rapid functional annotation by directly mapping raw reads to reference databases such as KEGG, SEED, and UniRef. For instance, FMAP is a straightforward and easy to use alignment based functional classification tool. It combines read mapping to a reference database, ortholog (KO) quantification, and statistical analysis all in one package (Kim et al. 2016b). In addition, HUMAnN2 employs a tiered strategy for efficient functional profiling based on alignment strategy: (i) rapid taxonomic identification using MetaPhlAn2; (ii) nucleotide-level read alignment against a sample-specific pangenome database for efficient gene assignment; and (iii) translated search of remaining reads against a broad protein database for comprehensive coverage (Franzosa et al. 2018). These methods substantially decrease computational demands by circumventing the assembly requirement. Although the alignment-based strategy has been widely used for bacterial functional interpretation in metagenomic studies, its application to the functional profiling of eukaryotic organisms is a substantial challenge due to some limitations.

The advantages of the alignment strategy lie in its exceptional efficiency. This approach avoids biases introduced by assembly errors, thereby providing a more conservative representation of the original expression signals. However, several limitations warrant consideration. First, annotation efficiency and specificity are compromised by read length constraints; short reads (e.g., 150 bp) typically cover only partial protein domains, resulting in insufficient annotation specificity for multi-domain fungal genes. Meanwhile, since conserved metabolic genes are shared between bacteria and fungi, read-based functional annotations cannot distinguish their biological origins. This means a glycosyl hydrolase read could originate from either a fungus or a bacterium, thereby confounding ecological interpretation (Lind and Pollard 2021). Furthermore, an alignment-based analysis of unassembled reads using a MEGAN pipeline yielded fewer differentially expressed genes than an assembly-based approach in a wheat rhizosphere metatranscriptomic study (Hayden et al. 2018). Notably, read-based methods predominantly detect dominant taxa while overlooking low-abundance eukaryotic genes. Consequently, unassembled-read approaches exhibit a systematic bias toward bacterial genes and frequently assign fungal transcripts to “unassigned” or “hypothetical” categories, limiting their utility for detailed functional exploration of fungal systems (Stewart et al. 2011).

### Ecological applications of functional annotation

Functional annotation has been widely employed to elucidate the roles of fungal communities in ecosystems. For instance, in nutrient cycling dynamics studies, *in situ* metatranscriptomic analysis revealed seasonally shifting metabolic strategies in the deep-sea microbiome (He et al. 2025). Specifically, with this technique, the authors revealed the metabolic activities of active microbial groups and characterized the microbial contribution to element cycling in the deep ocean. In an analysis of symbiotic and pathogenic mechanisms, Wang et al. (2025) revealed that the fungus *Halophilomyces hongkongensis* engages in positive interactions with *Vibrio* bacteria, collaboratively participating in nitrogen fixation and lignin degradation in seagrass root systems.

Those cases demonstrate that assembly-based approaches are particularly suitable for in-depth mechanistic investigations using metatranscriptomic sequencing, such as discovery of novel secondary metabolites or functional differentiation studies at the species level. Conversely, alignment-based strategies are better suited for large-scale screening and macroscopic ecological pattern analysis, such as functional *β*-diversity assessment across different habitats. However, a fundamental challenge remains, the incompleteness of fungal databases limits both functional classification strategies. To address this, a systematic expansion of fungal protein family databases, coupled with integration of taxon-specific genes and metabolic pathways, would significantly improve the mapping efficiency of sequencing reads to fungal-specific functional units. For instance, incorporating fungal carbohydrate-active enzyme (CAZy) families into analytical pipelines enables effective identification of polysaccharide degradation potential in microbial communities (Abot et al. 2016). Similarly, specialized tools such as antiSMASH (fungal version) and SMURF can detect secondary metabolism genes (e.g., polyketides and non-ribosomal peptides) overlooked in general databases, thereby revealing unique fungal metabolic activities (Blin et al. 2021; Khaldi et al. 2010). In addition, machine learning models specifically trained on fungal sequences can overcome limitations of homology-based functional annotation. Specifically, deep learning frameworks adapted to fungal genomic features could be developed, establishing fungal-specific Hidden Markov models or neural network classifiers for gene families such as CAZymes and effector proteins, enabling accurate inference of unknown gene functions (Riedling et al. 2024).

## Current challenges and solutions in the bioinformatic analysis of fungal metatranscriptomics

The rapid advancement of fungal metatranscriptomics has significantly promoted research in fungal taxonomy and functional annotation, thereby enabling more precise species identification and a deeper understanding of the metabolic processes and functions performed by fungi in various ecosystems. However, the field continues to face methodological challenges that not only limit the depth of data interpretation but also compromise the reliability of research conclusions.

### The complexity of fungal transcriptomes

Fungal transcriptome complexity presents multidimensional challenges, encompassing expansive and repetitive genomes, intricate post-transcriptional regulation, and significant environmental background interference. For example, rust fungi (*Pucciniales*) have an average genome size of 300 Mb (ranging up to 900 Mb) and are characterized by a high content of repetitive genes, transposable elements, and introns (Cristancho et al. 2014; Orozco-Arias et al. 2022; Tavares et al. 2014). In addition, most fungal genes contain introns and exhibit widespread alternative splicing. For instance, *Magnaporthe oryzae* generates 4,270 alternatively spliced isoforms from 2,413 genes, with approximately one-third only detected during host infection (Jeon et al. 2022; Zhong et al. 2020). Furthermore, fungal RNA typically represents only a minor fraction (often <5%) of total RNA in environmental samples, while host (e.g., plant roots) and bacterial RNAs constitute the major background noise (Amorim-Vaz and Sanglard 2016; Martin et al. 2024). Co-extracted environmental inhibitors such as humic acids and phenolic compounds further compromise RNA quality and interfere with downstream enzymatic reactions (Wang et al. 2012).

Long-read sequencing technologies provide an effective approach to address genomic and transcriptome complexity. Platforms such as PacBio enable direct acquisition of full-length transcripts, permitting accurate resolution of diverse fungal transcripts (Gordon et al. 2015). For instance, the Iso-seq technology developed by PacBio demonstrated critical utility in refining eukaryotic gene annotation, as exemplified by the *Zymoseptoria tritici* genome (Lapalu et al. 2025). By providing full-length transcript sequences, it precisely delineated 5’ and 3’ UTR boundaries for 73% of the re-annotated gene models and uncovered prevalent alternative splicing mechanisms, primarily intron retention, which were previously unresolved (Lapalu et al. 2025). These improvements substantially elevate the accuracy and completeness of both alignment- and assembly-based analyses of complex fungal transcriptomes. For external genetic contamination, optimized RNA extraction and enrichment have demonstrated considerable efficacy. Probe-based fungal rRNA removal techniques enhance fungal mRNA sequencing efficiency compared to the Illumina Ribo-Zero Plus Kit (Hu et al. 2022). Furthermore, the combined use of phosphate buffer and acidic sodium acetate buffer during bead-beating and phenol-chloroform extraction processes substantially enhances both RNA yield and integrity from soil samples (Hu et al. 2022). Collectively, these methodological advances significantly improve the reliability and validity of downstream transcriptomic data, providing support for precise detection of fungal transcriptomes in complex environmental samples.

### The scarcity and bias of reference databases

Current fungal reference databases exhibit substantial biases in species coverage and functional annotation completeness. Over 80% of sequenced fungal genomes fall within the *Ascomycota* and *Basidiomycota* phyla, while early-diverging lineages (such as *Zoopagales* and *Blastocladiomycota*) remain severely underrepresented (Gryganskyi et al. 2023; Muggia et al. 2020). This systematic bias results in the absence of closely related reference sequences for numerous environmental fungal transcripts, forcing reliance on distant homology-based strategies during annotation that frequently lead to functional misassignment. For example, incomplete reference databases in *Grifola frondosa* resulted in the failure to identify numerous secondary metabolism genes (Song et al. 2022). This issue commonly extends to novel transcripts with no homology matches and leads to the inaccurate mapping of divergent genes to non-specific KEGG pathways, which significantly reduces functional insights.

Large-scale international genome sequencing initiatives are progressively addressing these limitations. The JGI 1000 Fungal Genomes Project aims to systematically sequence representative species across the fungal tree of life, with over 3,800 high-quality genomes currently released (Grigoriev et al. 2014). Concurrently, specialized databases continue to expand, providing a more comprehensive foundation for functional annotation. FungiDB integrates genomic, transcriptomic, and functional annotation data from more than 1,000 fungal species, achieving a 40% higher Gene Ontology (GO) annotation coverage compared to general-purpose databases (Basenko et al. 2018). The MycoCosm platform specifically focuses on environmentally relevant fungi, incorporating extensive genomic data from plant symbionts and soil saprotrophs (Ahrendt et al. 2022). Comparative studies confirm that fungal-specific databases dramatically enhance annotation sensitivity compared with other tools.

### Limitations in analysis workflow and tools

Fungal metatranscriptomics currently lack standardized analytical workflows, with significant variations in tool combinations and parameter settings across studies severely compromising the comparability and reproducibility of results (Anwar et al. 2019). Most general-purpose bioinformatics tools were originally developed for bacterial or model eukaryotic organisms and thus exhibit limited adaptability to fungal-specific characteristics (Li et al. 2015). For example, the widely used assembler metaSPAdes shows an approximately 25% increase in error rates when processing fungal genomic regions with high GC content, while Kraken2 demonstrates false positive rates as high as 15% in species-level identification for certain fungal lineages (Nurk et al. 2017).

The development of fungal-specific tools represents a critical pathway to addressing these challenges. FunOMIC integrates fungal-specific marker genes and protein databases, enabling simultaneous taxonomic classification and functional annotation, achieving 92% detection sensitivity for fungal species in benchmark evaluations (Xie and Manichanh 2022). MicroFisher employs specialized databases constructed from highly variable fungal genomic regions, significantly improving species-level resolution (Wang et al. 2026). The introduction of machine learning methods offer new opportunities for enhancing annotation capabilities. The deep learning-based gene prediction tool FunGenePred, trained on fungal-specific models, has improved ORF prediction accuracy by 18% (Zhang et al. 2022). Concurrently, the adoption of containerization technologies has facilitated the standardization of analytical workflows. Pipeline management tools such as Nextflow and Snakemake have simplified the deployment and reproducibility of complex analytical processes (Ghimire et al. 2025; Webb et al. 2024). Community-driven benchmarking initiatives, such as the Comparative Analysis of Metatranscriptomics (CAMI), are promoting continuous tool improvement and establishing best practices by providing standardized test datasets and evaluation metrics (Meyer et al. 2025).

## Conclusion

Metatranscriptomic technology is driving a significant shift in fungal ecology by simultaneously resolving “taxonomic identity” and “functional activity”. This approach not only overcomes the limitations in functional accessibility of traditional amplicon sequencing but, more importantly, enables *in situ* analysis of the ecological functions of fungal community. However, the field of metatranscriptomics still faces constraints; short-read sequencing is limited in resolving complex gene families, such as distinguishing members of the laccase multigene family in *Basidiomycota*. As well, the capture efficiency of fungal mRNA from environmental samples remains generally low, typically below 5% in soil samples, significantly impairing the detection of low-abundance functional genes. Functional annotation shows inadequate coverage of fungal-specific metabolic pathways, with over 60% of polyketide synthase genes lacking accurate annotation in KEGG database. Furthermore, the absence of standardized protocols further compromises study comparability, particularly regarding the determination of sequencing depth and functional normalization methods.

Tool innovations are poised to advance the field, integrating long-read sequencing technologies that hold promise for resolving transcriptional heterogeneity within fungal populations, while spatial transcriptomics enables *in situ* exploration of gene expression in tissue contexts. Methodologically, artificial intelligence is emerging as a pivotal tool, AlphaFold3 can accurately predict fungal enzyme structures of unknown genes, and deep learning tools have significantly enhanced the annotation of genes of unknown function. With continued technical advancements, fungal metatranscriptomics is transitioning from descriptive research to a predictive science, offering powerful support for understanding and managing fungal resources.

## Additional information

### Artificial Intelligence (AI) use

The authors accept full responsibility for the content of the manuscript, including the disclosure of any use of AI.

No AI tools were used in the preparation of this manuscript.

### Adherence to national and international regulations

All the fungal strains used in this study have been legally obtained, respecting the Convention on Biological Diversity (Rio Convention).

### Author ORCIDs

Wenxing Hu https://orcid.org/0009-0000-9232-0279

Jingfei Hu https://orcid.org/0009-0006-2093-7759

Zhen Wang https://orcid.org/0000-0002-1307-934X

Haihua Wang https://orcid.org/0000-0002-7410-2064

### Data availability

All of the data that support the findings of this study are available in the main text.
